# Comprehensive Analysis of TRP Channel–Related Genes in Patients With Triple-Negative Breast Cancer for Guiding Prognostic Prediction

**DOI:** 10.3389/fonc.2022.941283

**Published:** 2022-07-07

**Authors:** Haojie Zhang, Xiangsheng Zhang, Xiaohong Wang, Hongguang Sun, Changran Hou, Yue Yu, Song Wang, Fangxu Yin, Zhenlin Yang

**Affiliations:** ^1^ The Second Medical College, Binzhou Medical University, Yantai, China; ^2^ Department of Thyroid and Breast Surgery, Binzhou Medical University Hospital, Binzhou, China

**Keywords:** TNBC, prognosis, TRP channels, immune, bioinformatic algorithms

## Abstract

**Background:**

Triple-negative breast cancer (TNBC) is a special subtype of breast cancer. Transient Receptor Potential (TRP) channel superfamily has emerged as a novel and interesting target in a variety of tumors. However, the association of TRP channel–related genes with TNBC is still unclear.

**Methods:**

The The Cancer Genome Atlas (TCGA)-TNBC and GSE58812 datasets were downloaded from the public database. The differentially expressed TRP channel–related genes (DETGs) were screened by limma package, and mutations of the above genes were analyzed. Subsequently, new molecular subtypes in TNBC-based DETGs were explored by consensus clustering analysis. In addition, Lasso–Cox regression analysis was used to divide it into two robust risk subtypes: high-risk group and low-risk group. The accuracy and distinguishing ability of above models were verified by a variety of methods, including Kaplan–Meier survival analysis, ROC analysis, calibration curve, and PCA analysis. Meanwhile, CIBERSORT algorithm was used to excavate status of immune-infiltrating cells in TNBC tissues. Last, we explored the therapeutic effect of drugs and underlying mechanisms of risk subgroups by pRRophetic package and GSEA algorithm, respectively.

**Results:**

A total of 19 DETGs were identified in 115 TNBC and 113 normal samples from TCGA database. In addition, missense mutation and SNP were the most common variant classification. According to Lasso–Cox regression analysis, the risky formula performed best when nine genes were used: TRPM5, TRPV2, HTR2B, HRH1, P2RY2, MAP2K6, NTRK1, ADCY6, and PRKACB. Subsequently, Kaplan–Meier survival analysis, ROC analysis, calibration curve, and Principal Components Analysis (PCA) analysis showed an excellent accuracy for predicting OS using risky formula in each cohort (P < 0.05). Specifically, high-risk group had a shorter OS compared with low-risk group. In addition, T-cell CD4 memory activated and macrophages M1 were enriched in normal tissues, whereas Tregs were increased in tumor tissues. Note that the low-risk group was better therapeutic effect to docetaxel, doxorubicin, cisplatin, paclitaxel, and gemcitabine than the high-risk group (P < 0.05). Last, *in vitro* assays, Quantitative Real-time PCR (qRT-PCR) indicated that TRPM5 was significantly highly expressed in MDA-MB-231 and MDA-MB-468 cells compared with that in MCF-10A cells (P < 0.01).

**Conclusion:**

We identified a risky formula based on expression of TRP channel–related genes that can predict prognosis, therapeutic effect, and status of tumor microenvironment for patients with TNBC.

## Introduction

In 2020, the number of new cases of breast cancer reached 2.26 million (1). At the same time, breast cancer is the most common malignant tumor in women. Triple-negative breast cancer (TNBC) is defined as a type of breast cancer, in which Estrogen Receptor (ER), Progestogen Receptorv (PR), and Human Epidermal GrowthFactor Receptor 2 (HER-2) are all negatively expressed. In addition, TNBC is extremely aggressive and has a high rate of early recurrence when compared with other breast cancer subtypes (2). Despite the fact that chemotherapy cure many individuals with early-stage TNBC, the majority of these patients eventually develop metastases (3). As a result, novel biomarkers or risk stratification system are urgently needed to guide the prognosis of patients with TNBC.

A recent review has highlighted that Transient Receptor Potential (TRP) channel superfamily has emerged as a novel and interesting target for therapeutic intervention in the context of breast cancer (4). The TRP channel superfamily is defined by a six-transmembrane-segment structure that acts as a multi-mode sensor for a variety of stimuli (5). The superfamily is classified into seven families based on their sequence: TRPA, TRPC, TRPM, TRPML, TRPN, TRPP, and TRPV (6). In addition, a new member, TRPS, was identified by Himmel and colleagues (7). Expression and function of TRP channels are linked to cellular processes that drive cancer progression, such as cell proliferation, migration, invasion, and apoptosis, as well as drug sensitivity (8). In fact, TRP channels act as an ion channel, and its changes are significant in most tumors (9). It is important to note that not only are ion channels dysregulated in cancer but also their regulators, effectors, and other interacting genes expression are significantly altered (10).

Given above the evidence, we explored the prognostic value of TRP channel interactors (TRP channel–related genes) in TNBC. In addition, the related genes that may be involved in the progression of TNBC were comprehensively analyzed. In addition, we established a risk stratification system to analyze the survival probability of each TNBC patient, so as to avoid personalized follow-up and treatment for patients.

## Materials and Methods

### Datasets

The expression profile data and clinical information of patients with TNBC were obtained using TCGA (115 tumor and 113 normal samples) and Gene Expression Omnibus (GEO) database (GSE58812, 107 tumor samples). It is worth noting that GSE58812 dataset only contains survival data but no detailed clinical information. Moreover, mutation data about TNBC, such as copy number variation (CNV), single-nucleotide variation (SNV), and methylation data were also downloaded from TCGA database. In collection of TRP channel–related genes, gene set (Reactome_TRP_channels) from the molecular signatures database and another gene set (inflammatory mediator regulation of TRP channels) from the KEGG database. Last, 120 TRP channel–related genes were used for bioinformatics analysis.

### Mutation and Differentially Expressed Analysis

The mutation frequency and oncoplot waterfall plot of differentially expressed TRP channel–related genes (DETGs) in patients with TNBC were generated by the maftools package. The difference in TRP channel–related genes expression in tumor and normal tissues was identified using the limma package (P < 0.05; |logFC| > 1). We then constructed a protein–protein interaction (PPI) network for DETGs using the search tool: Genemania (11).

### Development and Validation of TRP Score for Estimating Survival Risk

We used both cohorts to validate the prediction performance, whereas one of the training set (TCGA-TNBC) was used to construct the prognostic model. First, we identified significant prognostic genes by univariate Cox regression analysis (P < 0.1). Subsequently, we used the glmnet package to perform LASSO regression and Cox multivariate regression for screening genes participating in TRP score formula. The formula for calculating TRP risk score is as follows: (gene 1 expression × coefficient) + (gene 2 expression × coefficient) + … + (gene n expression × coefficient). In addition, all cases were divided into two groups (the low-risk group or the high-risk group) according to the median of the TRP risk scores. The OS was compared between the two subgroups *via* Kaplan–Meier analysis. PCA analysis was performed in the stats package. The timeROC package was used to perform a ROC curve analysis. Last, we applied independent prognostic factors determined by multivariate Cox regression to construct a prognostic nomogram using rms package.

### Analysis of Immune Status and Drug Sensitivity

CIBERSORT algorithm (12) to calculate the proportion of different immune cell types based on the expression level of immune cell-related genes. The output results of 22 infiltrating immune cells were integrated to generate an matrix of immune cell fractions for analysis. In addition, we used pRRophetic package for prediction of clinical chemotherapeutic response from tumor gene expression levels (13).

### Cell Culture

The MDA-MB-231 cell lines were purchased from Nanjing KeyGen, and the MDA-MB-468 cell lines and MCF-10A cell lines were purchased from Wuhan Procell. MDA-MB-231 cells and MDA-MB-468 cells were cultured in L-15 medium supplemented with fetal bovine serum (FBS) and antibiotics. MCF-10A cells were cultured in special culture medium (Procell CM-0525). All cells were tested negative for mycoplasma and maintained in 5% CO_2_ at 37°C.

### Quantitative Real-Time PCR Analysis

Isolation of total RNA from the three cells (MCF-10A, MDA-MB-231, and MDA-MB-468) was performed according to the instructions for the TRIzol reagent, and the purity and concentration of RNA were determined by MV3000 Micro-Spectrophotometer (260/280 ratio). Equal amounts of RNA were then reversely transcribed to make complementary DNA. Then, we selected and designed primers. The qRT-PCR of TRPM5 was carried out using the primer set (forward: 5′-TTGCTGCCCTAGTGAACCAG-3′, reverse: 5′-GCACGATGTCCTCCCAAGAG-3′). In addition, we used Glyceraldehyde-3-phosphate Dehydrogenase (GAPDH) as an internal control gene; reactions were carried out using the SYBR Green Premix Pro Taq HS qPCR Kit (Accurate Biology, AG11701). Last, a Bio-Rad CFX96 Real-Time PCR Detection System was used for the determination of the target gene expression levels (TRPM5). Each sample was repeated three times. GAPDH was used as an internal reference gene. The relative abundance of each gene mRNA was calculated by the 2^−ΔΔCt^ method.

### Statistical Analyses

The statistical analyses were conducted in the R software (version 4.0.1). Specific statistical methods have been mentioned in the bioinformatics methods above. ***, **, *, and ns refers to P < 0.001, < 0.01, < 0.05, and not significant, respectively.

## Results

### Expression Landscape of TRP Channel–Related Genes in TNBC

**Figure 1:**
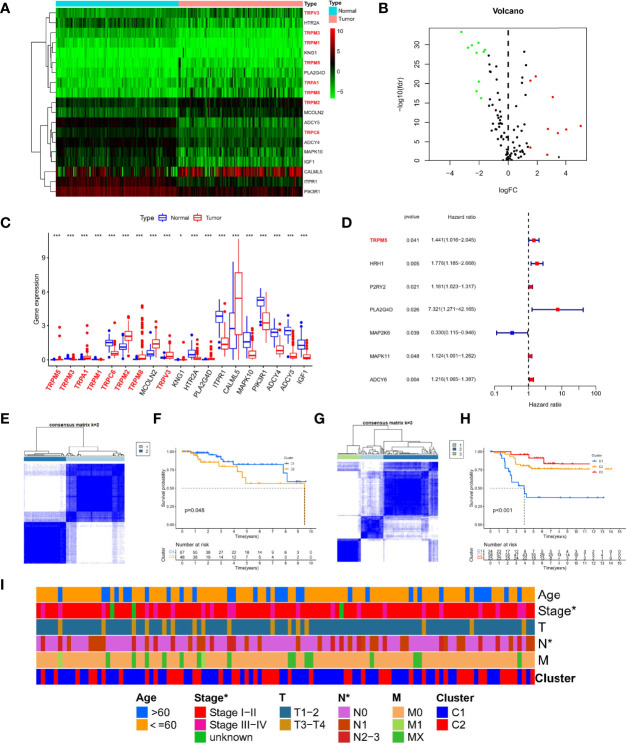
Expression landscape and molecular subtypes based on TRP channel–related genes. Heatmap **(A)**, volcano plot **(B)**, and boxplot **(C)** of expression of DETGs in tumor and normal samples. **(D)** A forest plot for the result of univariate Cox regression analysis in 120 TRP channel–related genes. **(E)** Consensus clustering analysis in the TCGA cohort based on different expression genes. **(F)** Survival analysis of two clusters in TCGA-TNBC. **(G)** Consensus clustering analysis in GEO cohort based on different expression genes. **(H)** Survival analysis of three clusters in TCGA-TNBC. **(I)** A composite heatmap containing clinical information in the TCGA cohort. *P < 0.05, and ***P < 0.001.

We obtained gene set (Reactome_TRP_channels) from the molecular signatures database and another gene set (inflammatory mediator regulation of TRP channels) from the KEGG database. Last, we collected 120 TRP channel–related genes for follow-up analysis. Using limma package to analyze RNA-seq data of TCGA-TNBC cohort, a total of 19 DETGs were identified in 115 TNBC and 113 normal samples (**Figure 1A**). The genes highlighted using red in the heatmap are members of the TRP superfamily, including TRPM5, TRPA1, TRPM2, TRPM8, TRPV3, TRPM3, TRPM1, and TRPC6. Moreover, nine genes were upregulated and 10 downregulated in tumors compared with normal samples (**Figure 1B**). Specifically, the nine upregulated genes were TRPM5, TRPA1, TRPM2, TRPM8, MCOLN2, TRPV3, KNG1, PLA2G4D, and CALML5. The 10 downregulated genes were TRPM3, TRPM1, TRPC6, HTR2A, ITPR1, MAPK10, PIK3R1, ADCY4, ADCY5, and IGF1 (**Figure 1C**). In addition, to further verify the prognostic ability of TRP channel–related genes (P < 0.05), univariate Cox regression analysis was used to screen. The results showed that only MAP2K6 was protective factor, whereas TRPM5, HRH1, P2RY2, PLA2G4D, MAPK11, and ADCY6 were risk factors (**Figure 1D**). Notably, in the TRP superfamily, only TRPM5 was differentially expressed in different samples and had prognostic value in TNBC. Therefore, we subsequently carried out *in vitro* assays on TRPM5 in TNBC cell lines.

### Different TRP Molecular Subtypes in Patients With TNBC

To explore the new subtypes in TNBC-based TRP–related molecular, we performed a consensus clustering analysis. In TCGA-TNBC cohort, the intragroup correlations were the highest, and the intergroup correlations were low when k = 2, indicating that patients could be well divided into two clusters based on 19 DETGs (**Figure 1E**). Similar, GEO-TNBC cohort (n = 107) were divided into three clusters when k = 3 (**Figure 1G**). Meanwhile, survival analysis showed significant differences in among different molecular subgroups (P < 0.05, **Figures 1F, H**). In addition, there were significant differences in stage and N staging among different subtypes in TCGA-TNBC cohort (**Figure 1I**). Taken together, the significance of TRP channel–related genes on the survival and tumor progression of patients with TNBC was demonstrated from another perspective.

### Mutation Analysis of TRP Channel–Related Genes

**Figure 2:**
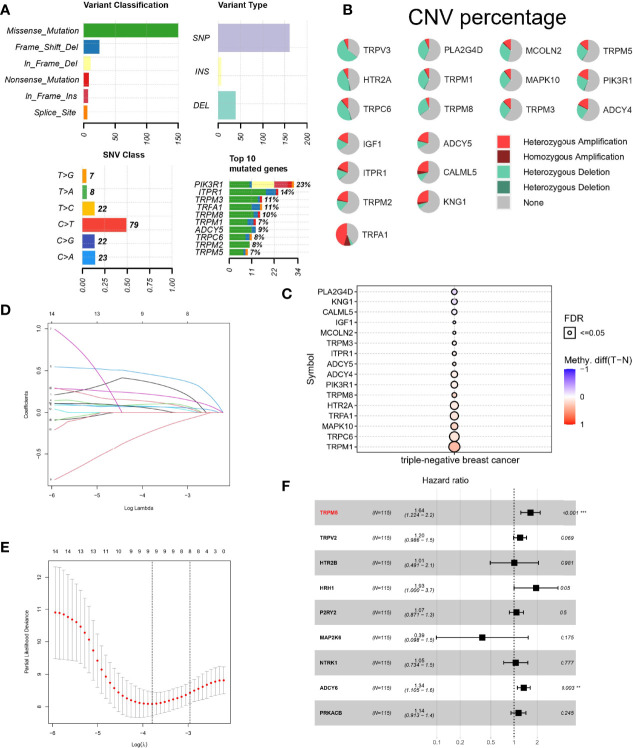
Mutation landscape and Lasso–Cox regression analysis of TRP channel–related genes. **(A**, **B)** Landscape of mutations about 19 DETRGs. **(C)** Methylation difference in different tissues. **(D**, **E)** LASSO regression. **(F)** A forest plot for the result of multivariate Cox regression analysis. **P < 0.01 and ***P < 0.001.

To further study the significance of TRP channel–related genes in TNBC, we downloaded mutation data from TCGA database and conducted in-depth analysis on above 19 DETGs. First, we performed a PPI network analysis of 19 DETGs and found that members of the TRP superfamily, including the genes mentioned above, were closely related to each other. Moreover, missense mutation was the most common variant classification. SNP were the most common variant type, and C>T ranked as the top SNV class. It is worth noting that the mutation frequency of PIK3R1 in the DETGs is the highest (**Figure 2A**). We also revealed the status of CNVs of 19 DETGs in patients with TNBC. The results showed that heterozygous amplification and heterozygous deletion were present in the vast majority of genes (**Figure 2B**). Last, we investigated the methylation of these genes with normal samples as controls. Interestingly, except for PLA2G4D, KNG1, CALML5, and IGF1, the other genes had more DNA methylation than the normal samples (**Figure 2C**). In addition, we used GSVA algorithm to estimate the role of each gene in cancer-related pathways, including apoptosis, cell cycle, DNA damage response, Epithelial-mesenchymal Transition (EMT), hormone AR, hormone ER, Phosphatidylinositol-3-kinase/AKT (PI3K/AKT), RAS/Mitogen Activated Protein Kinase (MAPK), Receptor Tyrosine Kinase (RTK), Tuberous Sclerosis Complex (TSC)/mechanistic Target of Rapamycin (mTOR). The results showed that TRP-related genes may activate most of above pathways, whereas role of inhibition only included a little pathways, such as cell cycle (**Figure S1**).

### Lasso–Cox Regression Analysis Based on TRP Channel–Related Genes

To calculate TRP score for estimating survival risk in patients with TNBC, 120 TRP channel–related genes were selected by univariate Cox regression analysis (P < 0.1), and the genes were further screened by LASSO regression analysis. The prognostic model performed best when nine genes were used (**Figures 2D, E**). Last, multivariate Cox regression analysis was used to calculate the regression coefficients of nine genes (**Figure 2F**). The formula of the model is as follows: TRP risk score = (−0.498711399 × expression level of TRPM6) + (-−0.15894075 × expression level of HTR2C) + (−0.217314244 × expression level of PLA2G4A) + (−0.690911672 × expression level of ASIC4) + (−0.955369151 × expression level of P2RY2) + (−0.949344105 × expression level of MAPK14) + (−0.699670637 × expression level of PLCG2) + (0.673798617 × expression level of SRC).

### Prognostic Efficacy of Risk Score Based on TRP Channel–Related Genes

**Figure 3:**
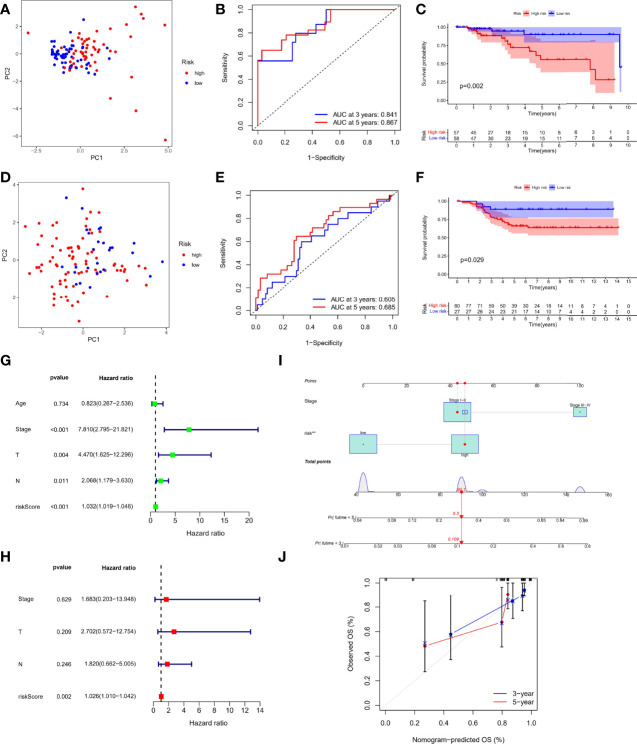
Prognostic efficacy of risk score based on TRP formula. PCA plot based on the risk score in the TCGA **(A)** and GEO cohort **(D)** Receiver Operating Characteristic (ROC) curves demonstrated the predictive efficiency of the risk score in the TCGA **(B)** and GEO cohort **(E)**. Kaplan–Meier curves in the high- and low-risk groups in the TCGA **(C)** and GEO cohort **(F)**. Univariate **(G)** and multivariate analysis **(H)** for the TCGA cohort. **(I)** Nomogram based on the TRP risk score and clinicopathological parameters. **(J)** Calibration curves of nomogram.

First, 107 patients from GEO cohort were utilized as the external validation cohort. According to the median score of TCGA-TNBC cohort, 115 patients in the TCGA cohort were divided into the low-risk group (n = 58) and the high-risk group (n = 57), whereas GEO cohort were divided into 80 patients as high risk and 27 patients as low risk, respectively. The PCA analysis showed satisfactory separation between the two risk subgroups (**Figures 3A, D**). ROC analysis of the TCGA-TNBC cohort showed that risk score had good predictive effect (AUC of 3 years = 0.841 and Area Under Curve (AUC) of 5 years = 0.867), as shown in **Figure 3B**. Similarly, it also showed excellent predictive ability in GEO-TNBC cohort (AUC of 3 years = 0.605 and AUC of 5 years = 0.685), as shown in **Figure 3E**. In addition, Kaplan–Meier survival analysis also indicated a significant difference in the OS between the low- and high-risk groups (P < 0.05; **Figures 3C, F**). Specifically, the high-risk group has a shorter overall survival time compared with the low-risk group.

### Independent Prognostic Value of the Risk Score

On the basis of RNA-seq combined with clinical information, we performed Cox regression analysis again to evaluate whether the TRP risk score could be used as an independent predictor of patients with TNBC. Univariate Cox regression analysis showed that risk score and other factors, including T staing, N staging, and clinical stage, were significantly correlated with OS (P < 0.05, **Figure 3G**) Multivariate Cox regression analysis showed that only risk score was independent risk factor associated with OS (P < 0.05, **Figure 3H**). In addition, considering the importance of clinical stage in clinical practice, we combined clinical stage and TRP risk score to construct the nomogram (**Figure 3I**), and the calibration curve showed good indicator performance (**Figure 3J**). Meanwhile, there were significant differences in TRP risk score among different clinicopathological factors, including age (P = 0.02, **Figure S2A**), clinical stage (P = 0.046, **Figure S2B**), and T staging (P = 0.03, **Figure S2C**).

### Comprehensive Immune Analysis

**Figure 4:**
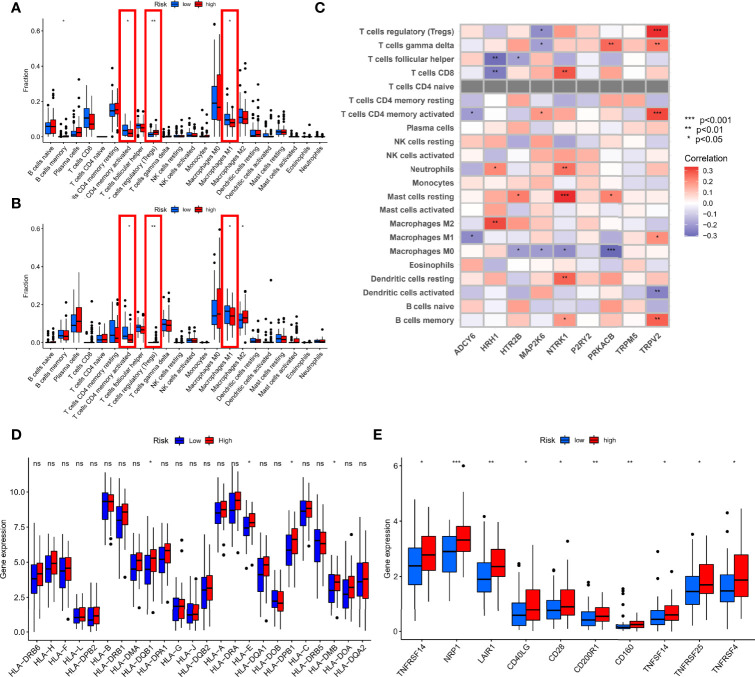
Comprehensive immune analysis. **(A)** Difference analysis of 22 types of immune cells in the TCGA cohort. **(B)** Difference analysis of 22 types of immune cells in GEO cohort. **(C)** Spearman analysis of 22 types of immune cells and each gene. **(D)** Difference analysis of mRNA expression of HLA. **(E)** Difference analysis of mRNA expression of checkpoints. *P < 0.05, **P < 0.01, ***P < 0.001 and ns, no statistical significance.

TNBC is the most immunogenic subtype of breast cancer, and there is strong evidence that tumor-infiltrating immune cells in TNBC have prognostic value and are associated with improved clinical outcomes (14). Therefore, we further comprehensively evaluated the guiding role of new subtypes based on TRP risk score in immunotherapy and immune-related mechanism. We explored the distribution of various immune cells, and the results showed macrophages accounted for a larger proportion of patients with TNBC in each cohort (**Figure 4A**). In the difference analysis using Wilcoxon test, boxplots showed that the three common types of immune cells in different tissues were significantly different (P < 0.05, **Figure 4B**). Specifically, T-cell CD4 memory activated and macrophages M1 were enriched in normal tissues, whereas Tregs were increased in tumor tissues. Meanwhile, Pearson analysis was used to explore the association of each gene participating in risk score formula with 22 types of immune cells. ADCY6 was negatively correlated with T-cell CD4 memory activated and macrophages M1 (P < 0.05). HRH1 was strongest positively correlated with macrophages M2 but strongest negatively correlated with T-cell follicular helper (P < 0.05). HTR2B was positively correlated with macrophages M2 but strongest negatively correlated with T-cell follicular helper (P < 0.05); other detailed correlation information was shown in **Figure 4C**. In addition, we explored the relationship between risk score and mRNA expression of HLA, immune checkpoints. In HLA analysis, only HLA-DQB1, HLA-E, HLA-DPB1, and HLA-DBM have difference in the different risk group, and high-risk group had a status of HLA overexpression (**Figure 4D**). Meanwhile, the boxplot showed only the immune checkpoints with differences (TNFRSF14, NRP1, LAIR1, CD40LG, CD28, CD200R1, CD160, TNFSF14, TMFRSF25, and TNFRSF4), and they were all upregulated in the high-risk group compared with the low-risk group, as shown in **Figure 4E**. Finally, HRH1 regulated most Transcription Factor (TF) and dominated the transcriptional regulatory network (**Figure S3**).

### Gene Set Enrichment Analysis in Different Risk Groups

**Figure 5:**
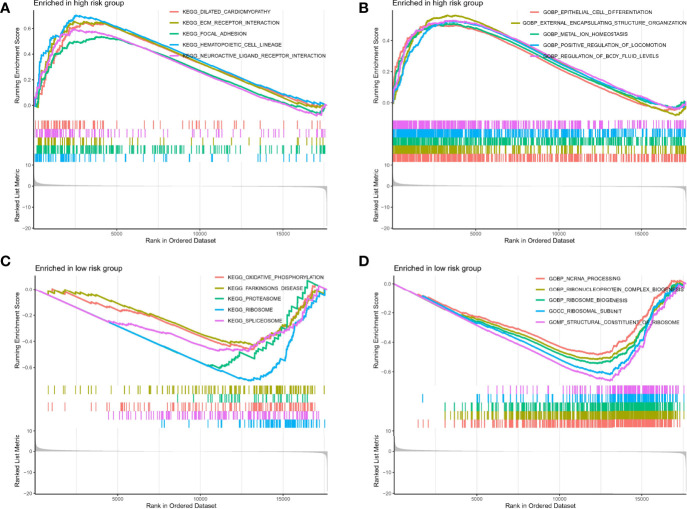
GSEA analysis. **(A**, **B)** GSEA analysis in high-risk group. **(C**, **D)** GSEA analysis in high-risk group.

To further explore the underlying mechanisms of different risks caused by disrupted TRP-related genes expression, we performed GSEA analysis to identify 672 Gene Ontology (GO) terms and 30 Kyoto Encyclopedia of Genes and Genomes (KEGG) pathways associated with different risk groups. As shown in **Figures 5A, B**, genes in the high-risk group were enriched in KEGG pathways, such as the ECM receptor interaction and focal adhesion, and GO terms, such as epithelial cell differentiation and metal ion homeostasis. In the meantime, as shown in **Figures 5C, D**, genes in the low-risk group were enriched in oxidative phosphorylation, Parkinson’s disease, non-coding RNA (ncRNA) processing, ribonucleoprotein complex biogenesis, and ribosome biogenesis.

### Prediction of Therapeutic Effect

**Figure 6:**
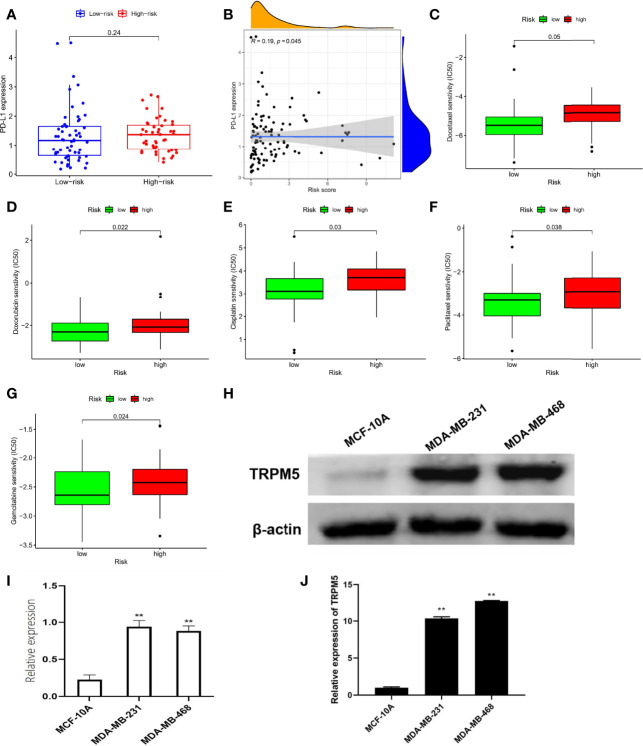
Prediction of therapeutic effect and expression validation. **(A)** Difference in PD-L1 expression. **(B)** Correlation analysis in PD-L1 with risk score. Difference in IC50 analysis about docetaxel **(C)**, doxorubicin **(D)**, cisplatin **(E)**, paclitaxel **(F)**, and gemcitabine **(G)**. **(H, I)** The protein levels of TRPM5 in MCF-10A, MDA-MB-231, and MDA-MB-468 were detected by Western blot. **(J)** The mRNA levels of TRPM5 in MCF-10A, MDA-MB-231, and MDA-MB-468 were detected by qRT-PCR.**P < 0.01.

In view of the important role of anti–PD-L1 agents and chemotherapy drugs in TNBC in the current clinical guidelines (15), we evaluated the therapeutic effect of drugs by pRRophetic algorithm. Although there was no difference in PD-L1 expression between different risk groups (**Figure 6A**), correlation analysis suggested a weak positive correlation: PDL1 expression increased with increased risk score (r = 0.19, P = 0.045, **Figure 6B**). Moreover, the results of IC50 were also interesting: Low-risk group was better therapeutic effect to docetaxel (**Figure 6C**), doxorubicin (**Figure 6D**), cisplatin (**Figure 6E**), paclitaxel (**Figure 6F**), and gemcitabine (**Figure 6G**) than high-risk group, suggesting the guiding role of new subtypes based on TRP risk score in chemotherapy.

### *In Vitro* Assays for a Hub Member of TRP Superfamily

Notably, in the TRP superfamily, only TRPM5 was differentially expressed in different samples and had prognostic value in TNBC (**Figure 1**), and it also participates in the construction of risk models (**Figure 2C**). Therefore, we made a bold hypothesis: TRPM5 has an underlying meaning in the occurrence and development of TNBC. Therefore, we conducted a preliminary experimental study on the expression of TRPM5. To further confirm the effects of TRPM5 on these cells, qRT-PCR and Western blot were performed. The results showed that the TRPM5 mRNA level of MDA-MB-231 and MDA-MB-468 cells was higher than that of McF-10a cells (P < 0.01), (**Figures 6H-J**).

## Discussion

Although there have been advancements in treating advanced TNBC, such as immunotherapy, there are still many difficulties for researchers and clinicians to conquer (16). After all, this is an advanced stage of the disease and improving the OS of such patients remains difficult. Therefore, the current goal is to research and develop biomarkers as soon as possible to predict the prognosis of patients with TNBC correctly (17). Ca2+ signaling is involved in many processes that affect the biological progression of cancer (18). Ion channels, especially TRP channel superfamily, are interesting situations for protein or gene expression because they regulate intracellular Ca2+ levels (19). Widespread dysregulation of TRP channel–related genes has been found in several types of cancers (20, 21), including breast cancer (22). Therefore, several TRP channel–related genes have been proposed as biomarkers of tumor progression and response to therapy in a variety of tumors. However, the association of TRP channel–related genes with TNBC is still unclear.

Although researchers have created many various approaches to define and quantify the immunological status of TNBC in contemporary studies (23, 24), attention to the prognostic role of widely expressed mechanosensitive calcium channels remains modest. In the study, we constructed a risky formula based on TRP-related genes. Patients were divided into groups according to high- and low-risk group, which had a value of indicting overall survival. In addition, most importantly, we also discussed the evaluation value in immune microenvironment and clinical application value of TRP risk score. In addition, we conducted *in vitro* experiments in TNBC cell lines on a hub member of TRP superfamily: TRPM5. Notably, TRPM5 also falls into members of the TRPM (“Melastatin”) family (25). TRPM5 mediates depolarization of the plasma membrane (26). Selective expression of TRPM5 was originally found in taste buds, suggesting that it may play a role in taste transmission (27). Meanwhile, TRPM5 is an intrinsic signal component of mammalian chemosensory organs (28). Research on TRPM5 in tumors is extremely scarce; although we conducted a simple experimental validation for TRPM5 in TNBC cell lines, further research is still needed in the future.

There are still some limitations of our study that are worth noting. First, the bioinformatics results, for starters, have been validated using TCGA and GEO samples. We were unable to conduct a second external validation, because we lacked the sufficient funding to sequence all patients with TNBC in our hospital. Second, we only used CIBERSORT algorithm to corroborate our findings for the association between status of immune microenvironment and risk score, and we will need to conduct more experiments in the future to confirm our conclusion except for only aiming at TRPM5. In conclusion, this study developed and validated a TRP risky formula that can guide clinical decision in TNBC.

## Conclusion

We identified a risky formula based on expression of TRP channel–related genes that can predict prognosis, therapeutic effect, and status of tumor microenvironment for patients with TNBC.

## Data Availability Statement

The datasets presented in this study can be found in online repositories. The names of the repository/repositories and accession number(s) can be found in the article/**Supplementary Material**.

## Author Contributions

HZ and XZ designed and conceptualized the study. XW and ZY supervised the study. HS, CH, and YY contributed toward data collection and analysis. All authors contributed to the article and approved the submitted version.

## Funding

This work was supported by Projects of Binzhou technology development program [No. 2015ZC0301], Scientific Research Staring Foundation of Binzhou Medical University [No. BY2014KYQD36, No. BY2014KJ36 and No. BY2017KJ01], Science and Technology Program of Universities in Shandong Province [No. J15LL51].

## Conflict of Interest

The authors declare that the research was conducted in the absence of any commercial or financial relationships that could be construed as a potential conflict of interest.

## Publisher’s Note

All claims expressed in this article are solely those of the authors and do not necessarily represent those of their affiliated organizations, or those of the publisher, the editors and the reviewers. Any product that may be evaluated in this article, or claim that may be made by its manufacturer, is not guaranteed or endorsed by the publisher.

## Supplementary Material

Supplementary Figure 1 GSVA algorithm to estimate the role of 19 DETGs in cancer-related pathways.

Supplementary Figure 2 A network of TFs-mRNAs.

Supplementary Figure 3 Differential analysis between clinical features and risk groups. Age **(A)**, stage **(B)**, and T staging **(C)**.
