# Supplementary figures and images for: Comprehensive Analysis of TRP Channel–Related Genes in Patients With Triple-Negative Breast Cancer for Guiding Prognostic Prediction
Source: Front Oncol. 2022 Jul 7;12:941283. doi: 10.3389/fonc.2022.941283 (PMC9300844; doi:10.3389/fonc.2022.941283)

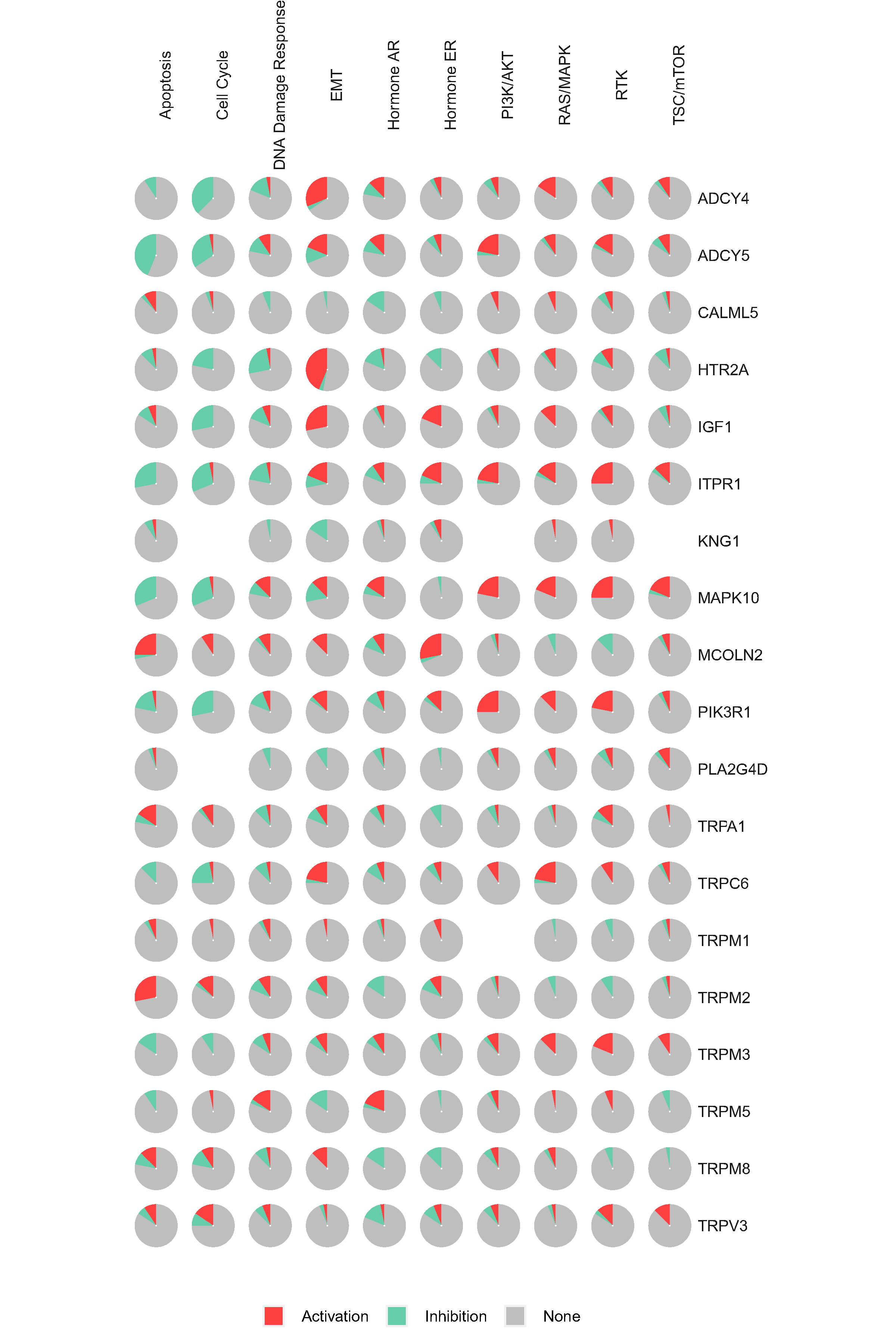

Supplement: Supplementary Figure 1 — GSVA algorithm to estimate the role of 19 DETGs in cancer-related pathways. [file Image_1.tiff]

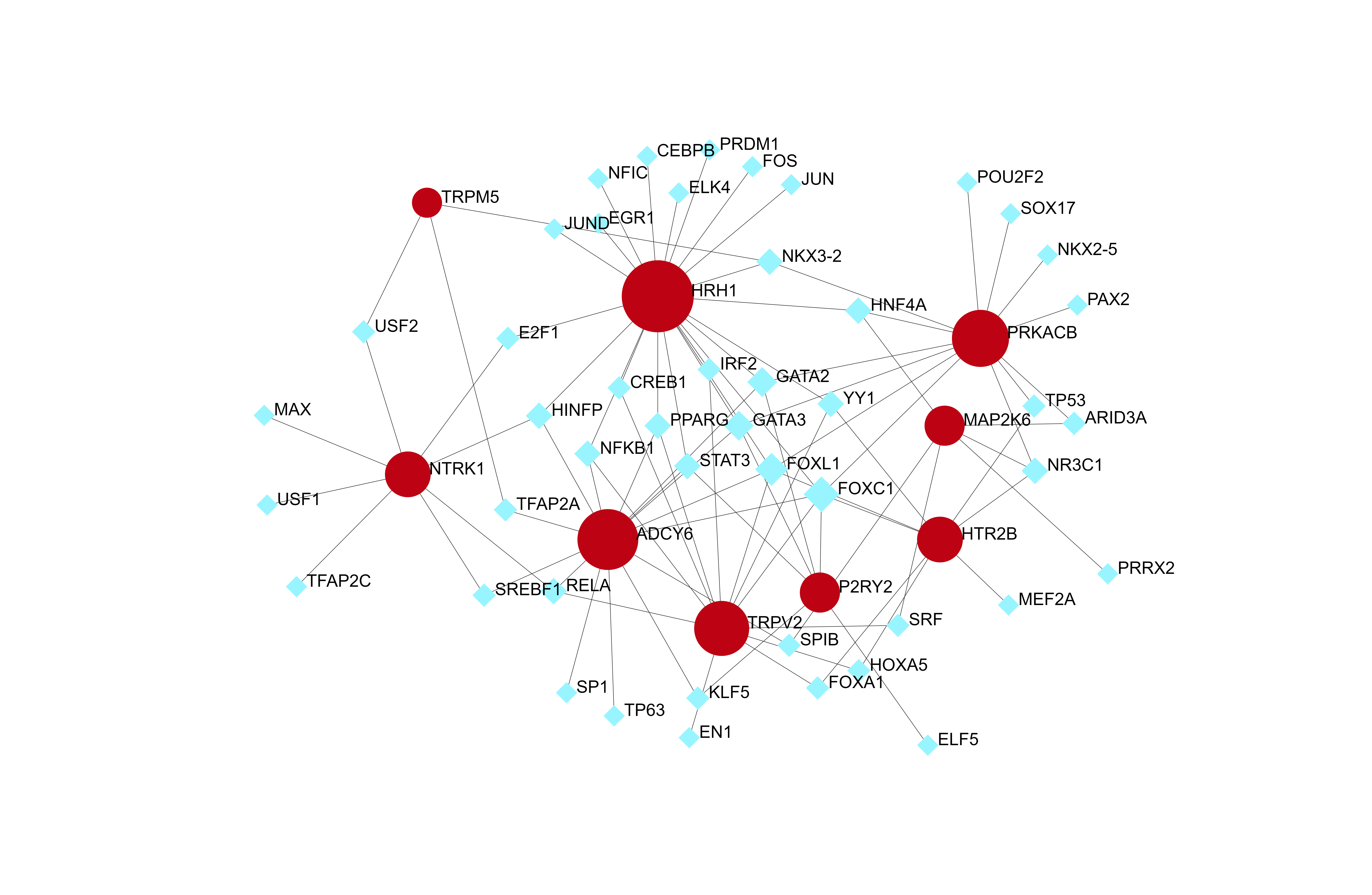

Supplement: Supplementary Figure 2 — A network of TFs-mRNAs. [file Image_2.png]

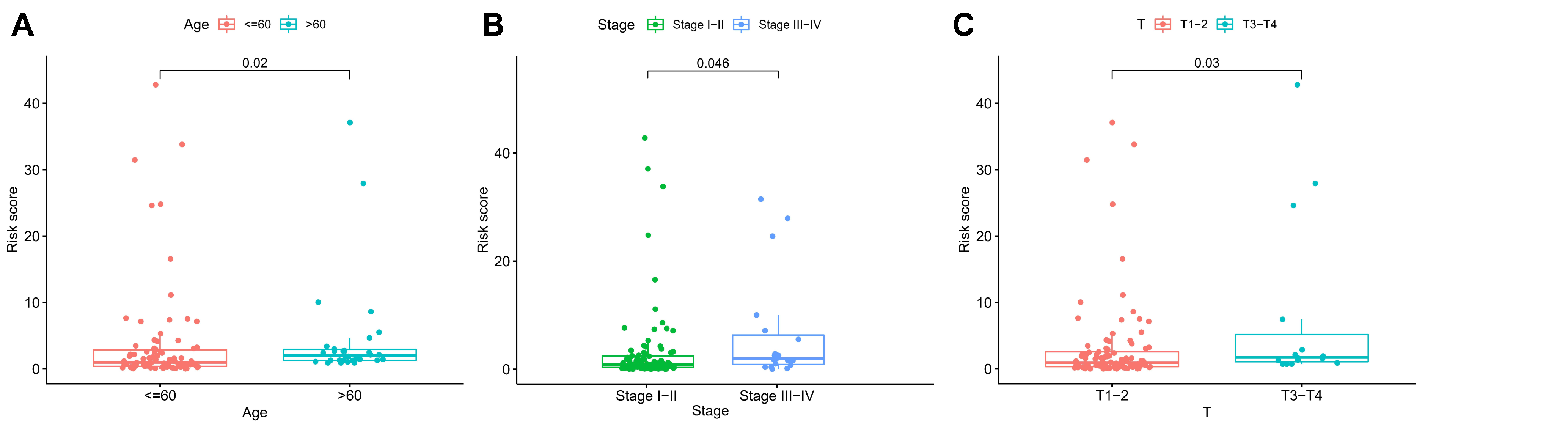

Supplement: Supplementary Figure 3 — Differential analysis between clinical features and risk groups. Age (A), stage (B), and T staging (C). [file Image_3.tiff]
